# Emotion and Feeling in Parent–Child Dyads: Neurocognitive and Psychophysiological Pathways of Development

**DOI:** 10.3390/children12111478

**Published:** 2025-11-02

**Authors:** Antonios I. Christou, Flora Bacopoulou

**Affiliations:** 1 Department of Special Education, University of Thessaly, 38221 Volos, Greece; 2 Clinic for Assessment of Adolescent Learning Difficulties, Center for Adolescent Medicine and UNESCO Chair in Adolescent Health Care, First Department of Pediatrics, Medical School, National and Kapodistrian University of Athens, 11527 Athens, Greece; fbacopoulou@med.uoa.gr

**Keywords:** emotion, feeling, parent–child dyads, emotion transmission, environmental sensitivity, emotion regulation, synchrony, developmental neuroscience, psychophysiology

## Abstract

Although widely used across disciplines, the terms emotion and feeling remain conceptually ambiguous, particularly within developmental science. Emotion is defined as an evolutionarily conserved, biologically embedded system of action readiness and intersubjective communication, shaped by attentional, neural, and physiological reactivity to environmental salience. In contrast, feeling is conceptualized as the consciously experienced, representational outcome of emotional activation, emerging through cognitive appraisal and symbolic processing. Building upon this distinction, the review explores how emotion develops within parent–child dyads through coregulated neurocognitive and psychophysiological mechanisms. Drawing on empirical evidence from eye-tracking studies of visual attention to emotional faces, functional near-infrared spectroscopy (fNIRS) research on social-emotional activation in prefrontal brain regions, and cortisol-based assessments of hormonal synchrony, the paper highlights how emotional attunement and transmission are embedded in early caregiving interactions. The review also emphasizes the moderating role of environmental sensitivity—both in children and parents—in shaping these developmental pathways. By positioning emotion as a dynamic, intersubjective process and feeling as its emergent experiential correlate, this review offers a novel developmental framework for understanding affect and proposes directions for future research on resilience, dysregulation, and intervention.

## 1. Introduction

Emotional development is often complicated by ambiguity in distinguishing ‘emotion’ from ‘feeling,’ which are frequently conflated despite being theoretically distinct. Although often used interchangeably, these constructs represent interdependent but distinct processes. Emotion can be conceptualized as an evolutionarily conserved system of action readiness and intersubjective communication, whereas feeling refers to the consciously experienced, representational correlate of emotional activation shaped through appraisal and symbolic processing [1,2]. Clarifying this distinction provides a foundation for tracing how affect is transmitted, regulated, and internalized during child development.

Foundational theories highlight different dimensions of emotion. LeDoux described subcortical circuits that enable rapid survival-oriented responses, positioning emotions as biologically primitive mechanisms [3]. Barrett’s theory of constructed emotion, by contrast, emphasized the predictive role of cortical appraisal and conceptual knowledge in shaping differentiated feelings [4]. Tronick’s dyadic expansion of consciousness framework extended these debates into the relational domain, underscoring how emotions are co-created through caregiver–infant exchanges [5]. Collectively, these perspectives emphasize the need to integrate biological, cognitive, and relational views of emotional development.

Although the terms “emotion” and “feeling” are often used interchangeably in developmental science, multiple theoretical frameworks emphasize their distinction. Classic and contemporary accounts converge in viewing emotion as an embodied, psychophysiological response to salient stimuli, and feeling as the conscious, subjective awareness of those responses [6,7,8,9,10,11]. To ensure terminological clarity, Table 1 summarizes the definitional differences between emotion and feeling across neural basis, consciousness, temporal unfolding, measurement, and developmental considerations, drawing on both foundational and recent perspectives.

### Distinguishing Emotion and Feeling: Contemporary Perspectives

Beyond the foundational views of LeDoux, several contemporary frameworks in affective neuroscience and developmental psychology further clarify the emotion–feeling distinction. Neurobiological models such as Damasio’s somatic framework define emotions as complex “action programs”—bodily and neural responses triggered by salient stimuli to maintain homeostatic balance—whereas feelings are the conscious mental representations that arise when the brain interprets those bodily changes. In this view, encountering a threat elicits an automatic emotional response (e.g., autonomic arousal, fight-or-flight behavior), and the subjective feeling of fear emerges only when cortical networks integrate the physiological changes into a coherent conscious experience [6,7]. Likewise, affective neuroscience research (e.g., Panksepp’s theory) distinguishes between evolutionarily “primary-process” emotions, which are generated by subcortical circuits and can operate without cortical input, and higher-order feelings that depend on cortical representation and self-awareness [15]. For example, decorticate animal studies and cases of human infants with minimal cortex demonstrate that basic emotional reactions (crying in distress, laughter in joy) are present even in the absence of full conscious awareness [15]. These findings suggest that core emotional processes are “built-in” subcortical functions, whereas feelings require additional integrative processes in the brain’s cortex to become subjectively accessible.

Developmental perspectives similarly emphasize that emotions precede feelings in ontogeny. Infants express basic affective states from early in life (e.g., crying when distressed, smiling when pleased), but these raw emotions are initially not accompanied by reflective awareness. Only with the gradual maturation of cognitive capacities—such as self-recognition, memory, and language—do feelings (in the sense of conscious, representational experiences of emotion) begin to emerge. Developmental researchers note that while basic emotions like joy, anger, and fear appear within the first year of life, the second year marks a critical transition when toddlers gain self-awareness and symbolic thinking, enabling the first self-conscious feelings to manifest. For instance, classic observations indicate that emotions linked to self-reflection—embarrassment, empathy, pride, shame—only become evident around 15–24 months of age, once children can mentally represent themselves and their internal states. In line with this, contemporary developmental psychology frameworks (e.g., Sroufe’s organizational theory) propose that early emotional reactions gradually differentiate into more complex feelings as the child’s neural and cognitive systems mature [16]. Collectively, these perspectives converge in portraying emotion as an evolutionarily ancient, automatic and embodied response system, and feeling as its later-developing, conscious and interpretive counterpart. Emotion can thus occur without feeling (e.g., an infant’s startle or a conditioned fear response may activate even if the child cannot yet conceptualize “being afraid”), whereas feeling represents the conscious “story” the mind constructs about those emotion signals. Integrating these viewpoints—from Damasio’s neurobiological model to Panksepp’s cross-species findings and developmental evidence on when children first experience feelings—underscores that emotion and feeling, though deeply interrelated, operate at distinct levels of processing (automatic bodily reaction vs. reflective experiential insight). Recognizing this distinction provides a richer theoretical foundation for examining how parent–child interactions scaffold the progression from raw emotion to organized feeling in development.

Parent–child dyads are now recognized as the primary ecological context within which emotions are scaffolded into coherent feelings. Infants rely on caregivers’ facial expressions, vocal prosody, and touch for regulating arousal and assigning meaning to affective states [17]. Attachment theory provides a foundation for understanding this process, showing that secure early relationships facilitate emotion regulation and the internalization of coherent affective states, whereas insecure attachments heighten vulnerability to dysregulation [18,19,20]. Beyond attachment theory, early relationships can also be conceptualized through an object relations developmental perspective, which focuses on how internalized representations of the caregiver shape the infant’s emerging emotional life. As Ainsworth [21] noted, the infant–mother relationship involves both dependency and the gradual organization of object relations, through which the caregiver’s consistent responsiveness provides a template for emotional regulation and security. Within this view, the intersubjective experience of early dyadic exchanges—characterized by affective attunement, containment, and repair—forms the experiential matrix from which emotional expression and later feeling states emerge. These early interactions allow the infant to transform raw physiological arousal into organized affective meaning, strengthening the parent–child bond and laying the groundwork for coherent emotional self-representation.

Importantly, caregivers’ own mental health—such as maternal depression—can disrupt sensitive attunement and pose additional risk for child emotional dysregulation. The developmental bridge from emotion to feeling is forged within dyadic exchanges. Caregivers help regulate arousal, direct attention, and provide labels and narratives that confer meaning on otherwise raw physiological signals. Micro-analytic studies show that affect is co-constructed through moment-to-moment cycles of attunement, mismatch, and repair; successful repair predicts greater coherence in children’s subsequent emotional experience [22]. Attachment theory extends this view: secure relationships offer a “safe haven” and “secure base” that support integration of arousal into organized, self-relevant feeling states, whereas inconsistent, intrusive, or neglectful care undermines modulation and leaves emotions unrepresented or dysregulated [23,24].

From a neurobiological perspective, emotions recruit conserved circuits that orchestrate autonomic, behavioral, and attentional responses, enabling fast adaptation to threat, novelty, or reward. Contemporary models emphasize rapid subcortical pathways that permit swift detection and motor preparation prior to, or independent from, reflective awareness [25]. At the same time, predictive and inferential processes shape how bodily states are categorized and used to guide behavior, highlighting the interactive layering of bottom-up reactivity with top-down expectations [26]. In early development, these functions are evident in cries, smiles, and gaze shifts that signal needs, elicit caregiving, and organize proto-conversations, thereby establishing a communicative platform on which later socioemotional competencies are built [27,28].

Feelings, by contrast, involve the conscious registration and interpretation of affective states. They emerge when interoceptive changes are integrated with memory, language, and conceptual knowledge to produce a subjectively accessible representation of “what it is like” to be in a particular state. Classic neurocognitive accounts propose that feelings arise as somatic changes are mapped onto cortical representations, supporting awareness and reportability of affect [29]. This distinction explains why emotions may occur without conscious recognition (e.g., rapid orienting to threat), whereas feelings require representational capacity, reflective access, and often linguistic scaffolding [30]. Because meaning systems vary across sociocultural contexts, feelings are also culturally shaped—constructed within the emotion concepts and norms available to the child [31].

In this review, emotion is framed as a biologically embedded system of action readiness—rapid, largely automatic, and tuned to environmental salience—whereas feeling denotes the consciously experienced, representational outcome of such activation, constructed through appraisal and symbolic processing [30,31]. In this view, the child’s movement from automatic reactivity to conscious feeling is a relational achievement, built through repeated co-regulated encounters that align attention, brain activity, and physiology with language and meaning.

The aim of this review is to provide a developmental framework that distinguishes emotion as an embodied, evolutionarily conserved process and feeling as its consciously experienced correlate, while situating both within parent–child dyads. Evidence is synthesized across multiple modalities: (a) attentional synchrony captured via eye-tracking, (b) neural synchrony examined with hyperscanning methods, and (c) hormonal synchrony assessed through cortisol coupling. By integrating these domains, the review positions emotional development not as an isolated individual process but as an emergent property of dynamic, multilevel caregiver–child interactions. Finally, the review emphasizes the importance of cultural and contextual influences. Cross-cultural research indicates that parental beliefs, caregiving practices, and sociocultural scripts shape how emotions are expressed and scaffolded into feelings [31]. Interventions designed to foster emotional attunement therefore require attention to both individual neurobiological sensitivity and broader sociocultural contexts. Through this synthesis, the review contributes theoretical clarity and outlines future directions for research on resilience, dysregulation, and clinical intervention.

## 2. Methodological Approach

A comprehensive literature search was conducted to identify peer-reviewed studies that examined the conceptual, neurocognitive, and psychophysiological bases of emotion and feeling within developmental contexts, with particular attention to parent–child dyads. Searches were performed in PubMed, Scopus, PsycINFO, and Web of Science covering the period from January 2000 to June 2025. Search terms combined free-text keywords and Medical Subject Headings (MeSH) related to emotion, feeling, parent–child dyads, synchrony, eye-tracking, functional near-infrared spectroscopy (fNIRS), cortisol, environmental sensitivity, and developmental neuroscience. An example PubMed search string was:

(“emotion” OR “affect” OR “feeling”) AND (“parent–child dyad” OR “synchrony” OR “attachment”) AND (“neurocognitive” OR “neurobiological” OR “psychophysiology”) AND (“development” OR “child” OR “infant”).

Reference lists of relevant systematic reviews and meta-analyses were also hand-searched to capture additional eligible studies [32,33]. Studies were included if they (a) involved child or adolescent participants up to 18 years of age, (b) reported empirical findings on emotional, neurocognitive, or psychophysiological outcomes in the context of parent–child interactions, and (c) were peer-reviewed and published in English. Exclusion criteria were case reports, editorials, conference abstracts, and studies focused solely on adult populations or purely biomedical outcomes without affective components.

Given the diversity of study designs, methodologies, and outcome measures, a narrative synthesis approach was employed rather than a quantitative meta-analysis. This strategy enabled integration across heterogeneous sources of evidence, ranging from neuroimaging and eye-tracking studies to psychophysiological measures such as cortisol synchrony. Findings were grouped according to key mechanistic domains, including attentional biases and visual scanning, neural coupling and cortical activation, and hormonal and autonomic synchrony. These domains were further embedded within relational frameworks such as attachment theory [34], dyadic regulation models [35], and predictive processing accounts of emotion [36,37].

Parent–child synchrony is shaped not only by biology and individual differences but also by the cultural contexts in which dyads develop. In collectivist societies, caregiving often emphasizes relational harmony, interdependence, and emotional restraint, whereas in individualist contexts, parents may encourage overt expression and autonomous self-regulation. For instance, a recent study comparing Palestinian-Arab (collectivist) and Jewish (more individualist) mother–infant dyads within Israel found that Arab mothers engaged in greater physical proximity and calming behaviors, while Jewish mothers engaged in more face-to-face play and active vocalization. Despite these differences in interactional style, infants in both groups demonstrated effective emotion regulation and secure attachment, suggesting that diverse caregiving strategies can achieve functionally equivalent socioemotional outcomes.

Cultural values also shape how “feelings” are conceptualized and labeled. In some East Asian contexts, feelings are construed relationally (e.g., maintaining group harmony), whereas in Western contexts they are often understood as internal, individualized states. Such differences mean that the trajectory from emotion (automatic physiological and behavioral responses) to feeling (conscious representation) may follow distinct cultural pathways. Over-representation of WEIRD (Western, Educated, Industrialized, Rich, Democratic) samples in synchrony research risks obscuring this variability. Greater cross-cultural research is needed to test whether existing models of dyadic synchrony and emotion–feeling transformation generalize beyond Western contexts, or whether multiple culturally specific routes to healthy emotional development exist.

While the literature reviewed is extensive, several methodological limitations remain. There is considerable heterogeneity in tasks, measurement tools, and cultural contexts, which constrains comparability across studies. Many investigations rely on small sample sizes or focus on specific populations (e.g., children at risk for psychopathology, children with neurodevelopmental disorders), limiting generalizability. Furthermore, cross-sectional designs dominate the field, leaving the developmental trajectories of emotion–feeling distinctions underexplored. These gaps highlight the need for harmonized, longitudinal, and cross-cultural research approaches that can more robustly capture how dyadic processes shape the emergence of emotional experience and conscious feeling across the lifespan [38,39,40,41].

In line with a narrative (qualitative) review approach, we broadly surveyed the literature rather than performing a formal meta-analysis. To increase transparency, we provide an overview of the scope of the literature search and synthesis. The initial database searches (across PubMed, Scopus, PsycINFO, and Web of Science for 2000–2025) yielded approximately 600–650 unique articles after removing duplicates. Titles and abstracts were screened for relevance to the topics of emotion, feeling, and parent–child developmental processes, resulting in roughly 150 articles retained for more detailed evaluation. Of these, about 70–80 studies met our inclusion criteria and were ultimately included in the narrative synthesis. These included empirical research spanning modalities (e.g., behavioral observations, eye-tracking, neuroimaging, hormonal assays) as well as theoretical and review papers that informed our conceptual framework. We emphasize that these figures are approximate, as is typical in narrative reviews, which do not adhere to the rigid counting of a systematic review. Nonetheless, providing this range offers readers a sense of the evidence base: on the order of a few hundred studies were surveyed, with a few dozen key sources integrated into our analysis. This narrative synthesis approach allowed us to integrate heterogeneous evidence and draw connections across studies without the constraints of a single quantitative metric. By qualitatively summarizing convergent findings (rather than statistically aggregating them), we aimed to capture the richness of research on parent–child emotional dynamics. Still, we acknowledge potential biases inherent in narrative reviews—for example, the possibility of overlooking some studies or weighting illustrative findings—and thus we strove for comprehensive coverage by hand-searching reference lists and including multiple theoretical perspectives. The estimated numbers of identified, screened, and included articles above further clarify the scope of our review process and enhance the methodological transparency of this work.

**Figure 1:**
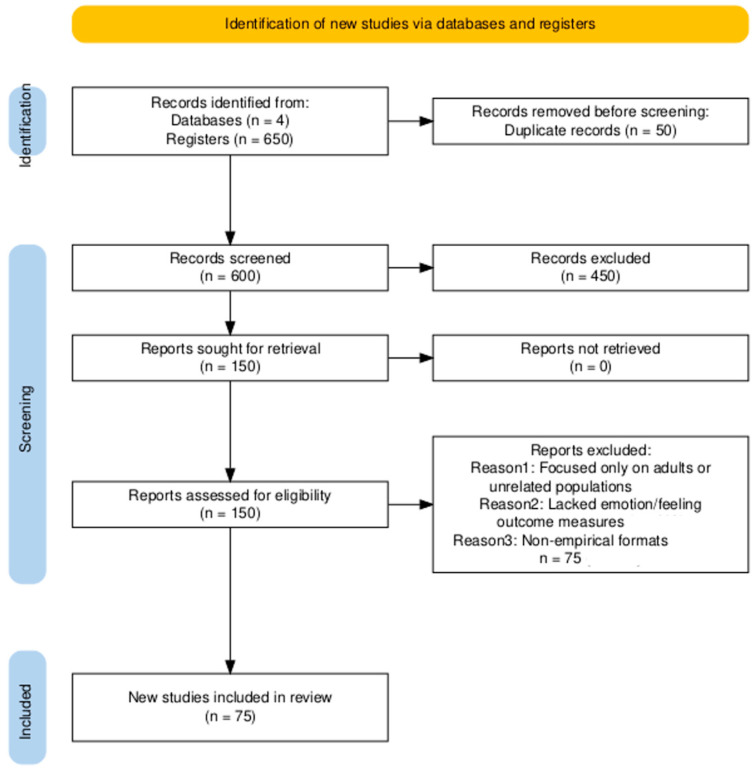
PRISMA-style flow diagram of study selection.

To further illustrate the study selection process and align with current reporting expectations, we have included a PRISMA-style flow diagram (Figure 1) summarizing the identification, screening, and inclusion of studies. While our approach is a narrative review rather than a formal systematic review, including this figure provides a clear overview of how studies were identified and selected. The flow chart outlines the number of records identified through database searches, the number remaining after screening and eligibility assessments, and the final set of studies included in the synthesis. Presenting the study flow in this familiar format is intended to enhance methodological transparency and demonstrate the rigor of the review process, even within a qualitative narrative framework.

## 3. Mechanisms of Dyadic Emotion Development

Parent–child dyads provide one of the earliest and most formative contexts in which emotion is scaffolded, regulated, and transmitted. Advances in developmental neuroscience and psychophysiology have enabled increasingly fine-grained accounts of how emotional attunement operates at multiple levels of analysis, from eye gaze to cortical activation to hormonal synchrony. This section synthesizes evidence across these domains, highlighting the multi-layered nature of dyadic emotion development.

### 3.1. Attentional Biases and Visual Scanning

Visual attention plays a central role in the early organization of emotion. Eye-tracking studies have consistently demonstrated that children display selective attentional biases to emotionally salient faces, particularly those expressing anger or fear [42,43]. These biases are not merely intrapersonal phenomena but unfold in the interactive space of the dyad. Parents’ attentional patterns shape and moderate how children orient to emotional cues, and congruency in parent–child gaze patterns predicts higher levels of affective attunement. Shared visual focus functions as a pathway of intersubjective communication and provides opportunities for emotion coaching [44,45].

Research by Pérez-Edgar and colleagues has been pivotal in documenting how early attentional biases emerge in infancy and contribute to trajectories of socioemotional functioning. For instance, infants at risk for behavioral inhibition display heightened vigilance to threat-related stimuli, with such attentional tendencies predicting later social withdrawal and anxiety [46,47]. Moreover, these attentional patterns are not fixed but embedded within the broader dyadic system. Quiñones-Camacho et al. emphasize that parent-to-child anxiety transmission unfolds through dynamic social interactions in which parental attention, affective responses, and regulatory strategies shape children’s emerging vigilance to threat [48]. From this perspective, attentional coordination between parent and child is one of the central pathways through which emotional dispositions are transmitted intergenerationally.

Recent longitudinal evidence further highlights the bidirectional nature of these processes. Vallorani et al. demonstrated that across the first 24 months of life, infants’ temperamental negative affect, maternal anxiety symptoms, and infants’ affect-biased attention are dynamically linked [49]. Infant negative affect predicts heightened threat vigilance, which in turn interacts with maternal anxiety to reinforce maladaptive attentional styles, whereas sensitive maternal regulation can attenuate such risk. These findings underscore that the congruence between parent and child gaze does not simply reflect shared focus but constitutes a training ground for attentional flexibility.

Parental sensory processing sensitivity may further amplify these dynamics: highly sensitive parents tend to attune more closely to their children’s scanning of negative affective cues, creating additional opportunities for emotional scaffolding and adaptive attention deployment [50]. Conversely, disruptions in attentional alignment—such as reduced parental responsiveness in depression or reinforcement of vigilance in anxious caregiving contexts—may limit opportunities for reciprocal regulation and heighten children’s risk for developing rigid attentional biases and anxiety-related psychopathology.

Recent studies further extend this evidence base. Aktar et al. [51] showed that even in infancy, parent–child dyads exhibit positively correlated attentional biases to emotional expressions, with both parents and infants orienting preferentially to fearful faces. Importantly, such alignment emerged even in low-risk families, suggesting that attentional synchrony is a fundamental feature of early dyadic interaction rather than only a marker of risk. In contrast, a 2024 eye-tracking study found that children of mothers with a history of depression displayed faster detection and longer dwell times for sad faces compared to controls, pointing to early attentional biases that may represent prodromal markers of affective vulnerability [52]. Taken together, these findings indicate that while parent–child attentional congruence can scaffold adaptive attention deployment and emotion understanding, it may also, in adverse contexts, reinforce maladaptive vigilance to negative cues.

Crucially, these findings also inform the distinction between emotion and feeling. The attentional coordination that characterizes early dyadic exchanges reflects the operation of emotion as a biologically embedded, intersubjective system of orienting to environmental salience. By contrast, the child’s later ability to consciously represent and interpret these affective encounters—as facilitated by parental scaffolding—marks the developmental emergence of feeling. Thus, research on attentional biases and parent–child gaze synchrony demonstrates how emotion initially manifests as shared, embodied orientation processes within dyads, and how the scaffolding of these attentional dynamics provides the experiential substrate from which subjective feeling states are eventually constructed.

### 3.2. Neural Coupling and Cortical Activation

Beyond attentional processes, parent–child dyads exhibit patterns of neural synchrony that support emotion regulation. Hyperscanning techniques such as functional near-infrared spectroscopy (fNIRS) and EEG hyperscanning have shown correlated prefrontal activation during joint tasks or affectively charged exchanges [53]. Such cortical coupling is not merely epiphenomenal: it has been linked to more adaptive emotion regulation, enhanced perspective-taking, and greater child social competence. For example, synchrony in the medial prefrontal cortex is associated with more effective parental scaffolding, reduced child distress in challenging contexts, and greater readiness to engage in cooperative problem solving [54].

Recent studies further underscore these associations. A longitudinal fNIRS study by Quiñones-Camacho et al. [48] demonstrated that higher parent–child neural synchrony in prefrontal regions during positive play predicted a faster decline in children’s internalizing symptoms over 1.5 years, suggesting that neural attunement serves a protective, resilience-building role. Similarly, Liu et al. [55] found that mothers generally exhibited stronger brain-to-brain synchrony with their 3–4-year-old children than fathers, particularly during shared activities such as joint video viewing. Importantly, both maternal and paternal synchrony decreased under conditions of high parenting stress, highlighting stress as a potential disruptor of neural attunement. These findings extend earlier work by showing that neural synchrony is sensitive not only to task context but also to caregiver well-being, and that it functions as a mechanism of resilience in supporting child socioemotional outcomes.

These results are consistent with predictive processing accounts of dyadic interaction [55]. From this perspective, parent and child continuously update internal models of the other’s emotional states, minimizing prediction errors via bi-directional neural signaling. Neural coupling thus provides a mechanism by which children learn to anticipate, interpret, and regulate affective states through live feedback with a caregiver. Reduced neural synchrony has been observed in dyads involving parents with psychopathology, highlighting the vulnerability of these mechanisms under stress.

In the context of this review’s central distinction, neural synchrony exemplifies emotion as an embodied, biologically instantiated process of intersubjective regulation. Shared cortical activation reflects the dynamic calibration of parent and child to one another’s affective states, forming the scaffolding for emotion as an action-oriented, communicative system. Over time, the repeated experience of such coupled neural states contributes to the child’s capacity to generate symbolic representations of affect—marking the emergence of feeling as the consciously experienced correlate of these dyadic exchanges. Thus, neural coupling represents both the mechanism through which emotions are co-regulated in real time and the developmental substrate from which children learn to consciously experience, label, and reflect upon their inner affective states.

### 3.3. Hormonal and Autonomic Synchrony

Physiological coupling provides another channel of emotional coregulation. Cortisol synchrony between parents and children has been consistently observed, particularly under conditions of stress, novelty, or emotional challenge [56,57,58]. Such biological linkage functions as a mechanism of affect transmission: when parents regulate their stress response effectively, children’s reactivity is buffered. Conversely, heightened or dysregulated parental cortisol responses may potentiate child stress and anxiety.

Recent work has nuanced this picture. Xu et al. [59] reported that the developmental impact of cortisol synchrony depends heavily on interactional context: synchrony predicted better child socioemotional adjustment under supportive, low-stress conditions, but showed diminished benefits in unsupportive environments. Similarly, Fleck et al. [60] (2023) found that in mother–adolescent dyads characterized by harmonious interaction, positive cortisol concordance was observed, whereas in high-conflict dyads or when adolescents exhibited borderline traits, synchrony was disrupted or negative. These findings highlight that cortisol alignment is not uniformly adaptive; rather, its meaning depends on whether it occurs in a regulatory or dysregulated caregiving context.

Autonomic indices further illustrate this biobehavioral attunement. Heart rate variability (HRV) synchrony, particularly vagal coupling, has been linked to effective co-regulation during emotionally demanding interactions, with higher synchrony supporting resilience and adaptive soothing [61]. Newer studies emphasize that vagal synchrony operates as a dynamic indicator of dyadic attunement. For example, high parent–child vagal synchrony has been associated with stronger child emotion regulation capacity and lower anxiety symptoms [62]. Conversely, disruptions in these processes—such as in contexts of maltreatment, inconsistent caregiving, or parental psychopathology—are associated with poorer physiological regulation and increased risk for psychopathology in children [63].

These findings underscore that physiological synchrony is both a marker and a mechanism of dyadic emotional functioning, mediating the pathway from caregiving quality to child developmental outcomes. They also reinforce that synchrony is context-dependent: in nurturing relationships, it provides a visceral sense of safety and scaffolds resilience, whereas in adverse contexts, it can transmit distress and dysregulation.

Framed within this review’s central conceptual distinction, hormonal and autonomic synchrony highlight emotion as a biological system of action readiness and intersubjective communication. Shared fluctuations in cortisol and vagal tone embody emotion’s role as an evolutionarily conserved process that organizes responses to environmental salience and transmits affective states across dyads. Over developmental time, the child’s repeated experience of these regulated physiological states, scaffolded by the parent, provides the somatic foundation from which feeling emerges as the conscious awareness of being soothed, distressed, or emotionally aroused. In this sense, physiological synchrony bridges the implicit bodily processes of emotion with the explicit subjective representations that constitute feeling, anchoring the experiential core of affective life in the parent–child relationship.

### 3.4. Integrative Dyadic Regulation Frameworks

The convergence of attentional, neural, and physiological synchrony supports broader theoretical accounts of dyadic regulation. Attachment theory has long emphasized the role of sensitive responsiveness in shaping secure emotional bonds and long-term socioemotional outcomes [63]. Tronick’s Mutual Regulation Model further highlights the inevitability of ruptures in dyadic attunement and the developmental importance of repair processes in fostering resilience and trust [64,65]. Feldman’s biobehavioral synchrony framework [66] extends these ideas by positing that moment-to-moment coordination across behavioral, neural, and physiological domains forms the basis for long-term adaptation. In parallel, environmental sensitivity (ES) models [67] add a complementary ecological layer, emphasizing that children differ in their susceptibility to environmental influences and that the quality of dyadic synchrony can amplify or buffer developmental trajectories. From this perspective, highly sensitive children are more strongly shaped by both supportive and adverse caregiving conditions, whereas less sensitive children may be less affected by contextual variation. ES models therefore situate dyadic processes within broader relational and sociocultural contexts, highlighting how individual differences in biological reactivity interact with parental scaffolding to shape emotion–feeling transformations.

**Figure 2:**
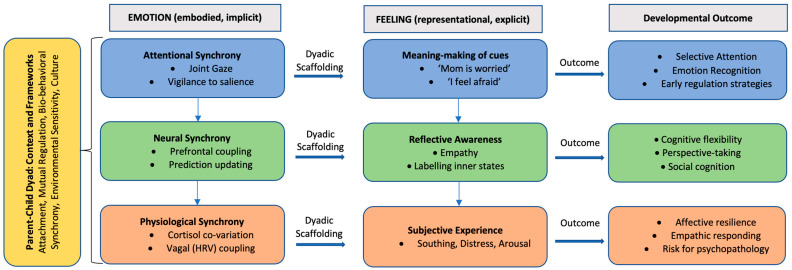
Conceptual model of how emotion (embodied, implicit) and feeling (representational, explicit) interact within parent–child dyads to shape developmental outcomes.

To integrate the reviewed evidence, we propose a conceptual model (Figure 2) that maps the interplay between emotion and feeling in parent–child dyads. Emotion is depicted as embodied, implicit processes of synchrony, encompassing attentional, neural, and physiological coordination. Through dyadic scaffolding, these implicit processes are gradually transformed into explicit forms of feeling, reflected in meaning-making, reflective awareness, and subjective experience. Each level of synchrony contributes to specific developmental outcomes: attentional synchrony supports selective attention, emotion recognition, and early regulatory strategies; neural synchrony facilitates cognitive flexibility, perspective-taking, and social cognition; and physiological synchrony underpins affective resilience, empathic responding, and vulnerability to psychopathology. By embedding these mechanisms within the broader parent–child context of attachment, mutual regulation, biobehavioral synchrony, environmental sensitivity, and culture, the model highlights how early intersubjective dynamics channel developmental trajectories toward adaptive or maladaptive outcomes.

When viewed through the distinction between emotion and feeling, these integrative models illustrate a developmental sequence. At the attentional level, joint gaze and shared vigilance toward emotional cues reveal emotion as a system of orienting and intersubjective signaling; over time, children’s internalization of these attentional patterns provides the groundwork for feelings as consciously accessible interpretations of those experiences. At the neural level, cortical coupling embodies emotion as a real-time, embodied process of co-regulation, while recurrent alignment of neural states supports the representational capacities that enable feelings of understanding, empathy, and self-awareness. At the physiological level, cortisol and vagal synchrony illustrate emotion as a biological readiness system, while repeated co-regulation of bodily states fosters the subjective recognition of being soothed or distressed—experiential correlates that constitute feeling.

While attachment, mutual regulation, and bio-behavioral synchrony provide universal frameworks for understanding how dyadic processes scaffold emotional development, environmental sensitivity frameworks highlight that children and parents differ systematically in their susceptibility to these processes. Emerging evidence suggests that such variability—captured in traits like sensory processing sensitivity, callous–unemotional tendencies, and neurodevelopmental profiles—modulates how the transformation from emotion to feeling unfolds. This rationale motivates a closer focus on individual differences in the following section.

## 4. Individual Differences and Environmental Sensitivity

Although atypical pathways are central to this review, it is equally important to outline normative trajectories of emotion and feeling. Research in developmental psychology and neuroscience shows that even in typically developing children, the capacity to experience, express, and regulate emotions undergoes rapid maturation in the first years of life [16,18,19]. From birth, infants display basic affective responses—distress when uncomfortable and pleasure when soothed—which act as biologically primed signals eliciting caregiving and serving adaptive functions [17]. During the first year, these undifferentiated states become increasingly organized: infants begin to show joy, surprise, anger, and fear, with wariness toward strangers emerging around 6–10 months, marking the transition from diffuse distress/excitement to discrete emotions [16].

By toddlerhood and preschool, children’s emotional repertoires expand considerably. The emergence of self-conscious emotions such as embarrassment, empathy, shame, and pride reflects the development of self-awareness, while growing language skills allow children to name and communicate feelings in simple terms [16,18]. By the end of the preschool years, most can anticipate others’ emotional reactions and use basic strategies—verbal labeling, seeking comfort, or covering their eyes—to manage fear or frustration. These advances parallel brain maturation, as increasing connectivity among prefrontal, limbic, and associative networks supports greater integration of emotion and cognition [25,26,27].

Caregiver–child interactions are critical to this progression. Sensitive and consistent caregiving provides external regulation in infancy, helping organize physiological arousal into coherent feeling states [17,18]. Through cycles of attunement, mismatch, and repair, children gradually internalize regulatory strategies and, by early childhood, those with warm, responsive caregiving typically display greater resilience and self-regulation [22,23,24]. Conversely, less attuned or inconsistent caregiving may complicate modulation of emotions, though normative milestones are generally achieved.

Individual differences temper these processes. Temperament traits evident from infancy shape how emotions are expressed and scaffolded: “easy” infants may regulate with minimal support, whereas more reactive or sensitive infants require greater caregiver involvement [16]. Such variability underscores that multiple healthy pathways exist and that there is no single trajectory of emotional development. What remains consistent, however, is that the first five years represent a critical period in which children move from reflexive, caregiver-managed states to increasingly autonomous and differentiated feelings. By school entry, most can identify basic feelings, empathize with peers, and verbalize experiences such as pride, guilt, or fear—laying the foundation for more complex emotional understanding in later childhood and adolescence [16,18,19].

Although dyadic synchrony provides a powerful scaffold for emotional development, not all children and parents engage with these mechanisms in the same way. Individual differences in temperament, neurocognitive profiles, and broader environmental sensitivity significantly shape how emotional exchanges are experienced and transmitted. This section highlights three key domains of variation: sensory processing sensitivity, callous–unemotional traits, and neurodevelopmental conditions, each of which can moderate the dynamics of parent–child emotional attunement.

### 4.1. Sensory Processing Sensitivity

Sensory processing sensitivity (SPS) is characterized by heightened reactivity to environmental stimuli, greater depth of processing, and elevated emotional responsiveness [68]. Within parent–child dyads, SPS has been linked to amplified attentional alignment, particularly in contexts involving emotional facial cues. Evidence suggests that children high in SPS display stronger gaze congruency with caregivers during exposure to negative affect, while parents with elevated SPS are more likely to co-orient to their child’s scanning patterns [69]. This bidirectional alignment may enhance opportunities for emotional scaffolding but also increase vulnerability to stress in overstimulating contexts [70]. SPS thus exemplifies a differential susceptibility mechanism, whereby heightened sensitivity can function as both a risk and resilience factor depending on environmental quality [71].

From the perspective of emotion and feeling, SPS amplifies the intensity of emotion as embodied synchrony (heightened gaze, physiological reactivity) while also accelerating or complicating the emergence of feeling as subjective awareness. In supportive environments, these children may more readily transform emotional activation into nuanced, reflective feelings, whereas in adverse environments, the same sensitivity may lead to overwhelming or dysregulated feeling states.

### 4.2. Callous–Unemotional Traits

Callous–unemotional (CU) traits, characterized by reduced empathy, shallow affect, and diminished responsiveness to others’ distress, represent another domain of variation with implications for dyadic synchrony. Studies indicate that children with elevated CU traits often exhibit attenuated gaze to emotional faces, reduced physiological synchrony, and weaker neural coupling during affective interactions [72]. These differences may limit the scaffolding opportunities that typically support the transformation of shared emotional states into reflective feelings.

Moreover, while CU traits are often viewed through a deficit lens, some evidence suggests that targeted interventions—particularly those emphasizing warmth, reward sensitivity, and explicit labeling of affective states—can partially restore synchrony and enhance reflective awareness [73,74]. Within the emotion–feeling framework, CU traits represent a condition in which the embodied foundations of emotion (synchrony, orienting, physiological co-activation) are disrupted, making the developmental leap to conscious feeling states less robust.

### 4.3. Neurodevelopmental Conditions

Neurodevelopmental conditions such as autism spectrum disorder (ASD) and attention-deficit/hyperactivity disorder (ADHD) further illustrate how structural differences in cognition and neurobiology modulate dyadic synchrony. Children with ASD may show atypical attentional alignment, including reduced gaze to faces or difficulties sustaining joint attention, which alters the channels through which emotional scaffolding occurs [75]. Similarly, altered neural coupling in ASD dyads may reflect challenges in prediction error minimization during social exchange [75]. In ADHD, heightened arousal and reduced regulation capacities can disrupt physiological synchrony (e.g., HRV coupling), complicating the establishment of stable co-regulated states [76].

Despite these challenges, interventions that scaffold alternative pathways of synchrony (e.g., multimodal cues, structured routines, or augmented communication strategies) can create new routes for bridging emotion and feeling. This highlights that dyadic scaffolding is flexible and that even when traditional synchrony mechanisms are disrupted, compensatory strategies may still enable the transformation of embodied emotional states into consciously represented feelings.

Taken together, these individual difference factors demonstrate that dyadic synchrony is not a uniform process but a contextually modulated system. Children with heightened sensitivity may thrive under supportive caregiving yet struggle under adversity, while those with CU traits or neurodevelopmental conditions may require tailored interventions to achieve similar developmental outcomes. Viewed through the emotion–feeling distinction, these variations underscore that highly sensitive children may experience amplified emotion that more readily transforms into nuanced or dysregulated feelings depending on context; children with CU traits may experience disrupted emotional scaffolding that weakens the consolidation of reflective feelings; and children with neurodevelopmental conditions may follow atypical synchrony pathways that require compensatory supports to foster the emergence of conscious feeling. Recognizing these patterns is essential for moving beyond one-size-fits-all models of emotion development and toward precision approaches that respect diversity in affective functioning.

## 5. Developmental Pathways and Risk for Dysregulation

Within parent–child dyads, synchrony across attentional, neural, and physiological systems provides the developmental bridge that allows emotion—an embodied, evolutionarily conserved system of action readiness—to be transformed into feeling, the consciously represented, narrativized correlate of emotional activation. When synchrony processes falter, children may continue to generate emotional reactions at the bodily and attentional levels yet fail to consolidate them into coherent, reflective feeling states. Environmental sensitivity frameworks clarify why disruptions do not affect all children equally: individuals higher in sensitivity (e.g., sensory processing sensitivity) are more responsive to both adverse and supportive contexts, magnifying risk under misattunement and resilience when caregiving is contingent and warm [77,78,79,80]. In this section, findings are explicitly parsed as emotion-level (automatic, embodied, synchrony-dependent) versus feeling-level (conscious, representational, language- and concept-dependent) phenomena.

### 5.1. Disruptions in Attentional Synchrony

Visual attention to emotional faces represents one of the earliest mechanisms through which emotions are organized in parent–child dyads. At the level of emotion, gaze serves as a rapid, embodied orienting response to environmental salience, allowing children to detect signals of safety or threat [81,82]. When caregivers and children fail to achieve congruency in their visual scanning patterns, the emotional signal is left unshared, reducing opportunities for dyadic coordination and intersubjective regulation.

Moving from emotion to feeling, this alignment provides the scaffolding through which raw attentional orienting is transformed into a consciously accessible interpretation of emotional meaning. In secure relational contexts, caregivers’ contingent responses and labeling practices help convert gaze-based reactions into felt experiences of comfort, threat, or affiliation [83]. In contrast, inconsistent or intrusive caregiving interrupts this process, leaving children with fragmented or poorly integrated affective experiences.

Notably, environmental sensitivity frameworks shed light on why disruptions in attentional synchrony do not affect all children equally. Children higher in sensory processing sensitivity show amplified responses to dyadic alignment: under supportive conditions, their heightened reactivity facilitates stronger co-orienting and richer emotional scaffolding, accelerating the consolidation of nuanced feeling states. Under adverse conditions, however, the same heightened sensitivity magnifies misalignment, resulting in overwhelming or dysregulated feelings [77,78,79,80].

### 5.2. Neural Dysregulation

Neural synchrony provides another layer of scaffolding through which emotion is shaped into feeling in parent–child dyads. Studies using fNIRS hyperscanning show that prefrontal coupling supports joint action readiness, sustained attention, and early co-regulatory processes during social interactions [84]. At the level of emotion, this shared cortical activation reflects a dynamic system for updating predictions and coordinating responses. When adversity such as parental stress or conflict disrupts this synchrony, the result is a breakdown in the alignment of emotional states between parent and child, leaving the child more vulnerable to unbuffered reactivity [83]. For this synchrony to be consolidated into feeling, cortical integration of interoceptive and contextual signals is necessary. When prefrontal coupling is preserved, it provides the substrate for reflective awareness, enabling children to represent and narrativize their internal states in ways consistent with Barrett’s predictive and conceptual models of feeling construction. When coupling is weakened, however, these processes stall, and emotions may remain raw, unprocessed reactions rather than evolving into differentiated, consciously accessible feelings.

Moreover, environmental sensitivity moderates this process. Children higher in sensory processing sensitivity are likely to experience larger swings in neural synchrony: when caregiving is attuned, synchrony is enhanced, promoting more elaborate reflective awareness and nuanced feeling states. In contrast, under misattuned or stressful caregiving, the same heightened sensitivity exacerbates disruptions, leading to stalled representational construction and dysregulated emotional development [78,79,80,81].

### 5.3. Altered Physiological Coupling

Parent–child synchrony is not limited to behavior or neural activity but also extends to the level of physiology, where shared rhythms in cortisol and heart rate variability (HRV) reflect the embodied transfer of arousal and regulatory tone. At the level of emotion, such coupling functions as a biobehavioral channel for transmitting stress and calm across the dyad. When synchrony is disrupted—for example, through mismatched cortisol peaks or reduced vagal concordance—children experience unbuffered emotional reactivity that undermines their capacity for self-regulation [85,86,87,88]. For these embodied dynamics to be consolidated into feeling, physiological states must be stabilized within the relational context so that bodily arousal can be integrated into an accessible narrative of safety, distress, or calm. When rhythms align under contingent caregiving, arousal is translated into coherent felt experiences (e.g., “I can calm down with you”), providing a physiological foundation for emotional meaning-making. When alignment fails, arousal remains fragmented and unintegrated, leaving children vulnerable to dysregulated or confusing affective experiences [87,88].

The environmental sensitivity framework highlights how this process is intensified in children and parents high in sensory processing sensitivity. In these dyads, physiological synchrony exerts greater “gain”: when caregiving is supportive, heightened sensitivity enhances co-regulation and enriches the child’s capacity to build nuanced feelings from emotional arousal. Conversely, when caregiving is inconsistent or stressful, the same sensitivity magnifies dysregulation, exacerbating the risk that emotions remain unprocessed and feelings poorly formed [78,79,80,81].

### 5.4. Developmental Cascade to Psychopathology

When disruptions in attentional, neural, and physiological synchrony converge, children are left with emotional reactions that remain poorly scaffolded and unintegrated. At the level of emotion, such convergent misalignments manifest as hypervigilance, heightened threat sensitivity, and weakened inhibitory control—patterns of raw reactivity that accumulate without the stabilizing influence of dyadic co-regulation [89]. Over time, these unbuffered responses create a developmental cascade in which children are more likely to respond rigidly or impulsively to emotional challenges. The absence of reliable scaffolding impairs the transformation of these embodied reactions into feelings, leaving emotions unrepresented, unlabelled, and uncontained. For some children, this produces internalizing trajectories, where persistent reactivity without reflective containment leads to anxiety or depressive symptoms. For others, the failure of emotional signals to be represented as morally encoded feelings of guilt, empathy, or concern contributes to externalizing trajectories, including aggression and callous–unemotional tendencies [90,91].

Within the environmental sensitivity framework, these divergent pathways are more clearly understood. Children high in sensitivity are particularly susceptible to the quality of their environment: under harsh or inconsistent caregiving, cascades are accelerated, leading to more severe dysregulation; under warm and predictable caregiving, the same heightened responsiveness allows for attenuation or even reversal of these risks [78,79,80,81]. This dual potential underscores how environmental conditions shape whether sensitivity operates as a vulnerability factor or as a source of resilience in the transition from emotion to feeling.

### 5.5. Protective and Compensatory Factors

Even in the presence of risk, protective processes within parent–child dyads can preserve the developmental bridge from emotion to feeling. At the level of emotion, sensitive caregiving stabilizes synchrony across gaze, neural coupling, and autonomic alignment, thereby reducing volatility in embodied responses and supporting co-regulation during stress [92,93]. These stabilizing dynamics ensure that children’s raw emotional arousal is contained within a relational context, preventing reactivity from becoming overwhelming. At the level of feeling, caregivers further scaffold the transformation of arousal into coherent experience by providing contingent labeling, narrative elaboration, and reflective dialogue. Through these symbolic and linguistic supports, children learn to interpret bodily states as subjectively meaningful feelings—fear, comfort, pride, or sadness—rather than as diffuse or confusing arousal. This scaffolding is especially beneficial for children high in environmental sensitivity, who show stronger positive returns in supportive contexts: under warm, predictable caregiving, their heightened reactivity facilitates the construction of more nuanced and differentiated feelings (so-called vantage sensitivity) [78,79,80,81,92,93,94,95].

Psychophysiological synchrony also functions as a protective mechanism when embedded in attuned caregiving. Aligned cortisol rhythms and vagal concordance amplify the translation of embodied arousal into accessible feeling states, acting as a biobehavioral “transducer” that supports resilience [96]. In this way, compensatory systems—whether located in caregiver warmth, sibling bonds, or peer relationships—operate not simply by suppressing emotion but by actively fostering the developmental processes that transform emotion into feeling.

Bringing together from the discussion above, environmental sensitivity operates as a gain-control parameter on the bridge from emotion to feeling. At the emotion level, sensitivity calibrates the magnitude of attentional capture, neural coupling, and physiological concordance; at the feeling level, it magnifies the effects of caregiving on the quality and granularity of conscious emotional representations. Under supportive dyadic conditions, high sensitivity accelerates the transformation of emotional activation into nuanced, reflective feeling; under adverse conditions, it accelerates dysregulated or blunted feeling (e.g., hyperaroused anxiety or reduced felt concern), clarifying apparently paradoxical outcomes within a single framework [79,80,81,82]. This account integrates earlier theory—rapid subcortical emotion circuits (LeDoux), predictive/conceptual feeling construction (Barrett), and dyadic scaffolding (Tronick; multilevel synchrony, Feldman)—into a unified, developmentally sensitive model that explains who benefits most from synchrony and when misattunement is most costly.

## 6. Integrative Developmental Framework

The evidence reviewed across attentional, neural, and physiological domains converges on a central insight: emotion and feeling do not emerge solely within the individual, but within the relational matrix of parent–child interactions. By distinguishing emotion as an embodied, evolutionarily conserved system of action-readiness and feeling as the subjective, representational correlate of this system, a clearer conceptual scaffold is provided for interpreting affective phenomena in development. Crucially, synchrony processes situate the dyad as the crucible of affective growth, building the bridge from raw, embodied reactivity to symbolic, consciously accessible experience. Within this framework, environmental sensitivity becomes a key moderator, determining whether synchrony processes consolidate into resilience or collapse into dysregulation.

Synchrony across attentional, neural, and physiological levels provides the foundation for transforming emotion into feeling. At the level of emotion, shared gaze patterns and attentional alignment function as rapid orienting mechanisms that prepare the child for action and signal salience in the environment [97,98]. At the level of feeling, caregivers translate these reactions into meaning through labeling, narrative, and symbolic scaffolds, allowing children to experience gaze-based orienting as consciously represented affect [99]. Similarly, neural coupling observed in fNIRS and hyperscanning paradigms reflects the co-construction of meaning at the emotion level, where dyads dynamically coordinate cortical activity during joint tasks [54,100,101]. This alignment enables the transition to feeling by providing a cortical substrate for reflective awareness and symbolic representation. Physiological synchrony, indexed by cortisol concordance and vagal tone coordination, represents an embodied channel through which arousal is shared and stabilized [102,103]. Under supportive caregiving, such physiological alignment becomes the basis for felt safety, demonstrating how emotional arousal can be converted into consciously accessible experience. Notably, these synchrony processes integrate dynamically: attentional scaffolds support neural coupling, which in turn sustains physiological co-regulation, creating a multi-level scaffold for the consolidation of emotion into feeling [104,105].

Synchrony mechanisms are not experienced uniformly. Children with high SPS display stronger amplification of synchrony signals, thriving in supportive caregiving environments but showing pronounced dysregulation when caregiving is inconsistent [106,107,108]. In such children, the movement from emotion to feeling is accelerated under positive conditions, producing nuanced and differentiated affective experiences, but is equally accelerated toward fragmentation under stress. Conversely, children with CU traits show attenuated attention to emotional cues and diminished synchrony across physiological systems [109,110]. In these cases, emotion signals may fail to trigger the embodied arousal necessary for feelings to be consolidated, leading to difficulties in moral encoding and empathic concern. Targeted interventions such as explicit affect labeling and warmth-based parenting can, however, partially restore synchrony and support the emergence of reflective feeling [111,112].

Neurodevelopmental conditions also illustrate how synchrony pathways can diverge. In autism spectrum disorder, atypical gaze and neural coupling patterns may alter the channels through which emotion is scaffolded, yet compensatory strategies—such as multimodal cues or structured routines—can still enable the translation of emotional activation into felt experience [54]. Attachment security further moderates these dynamics: secure relationships stabilize synchrony across levels, while insecure or disorganized attachment undermines the scaffolding of feelings from emotional states [113,114]. Taken together, these findings highlight that the transformation from emotion to feeling depends on the interplay between biological sensitivity, caregiving quality, and attachment context, shaping trajectories of both vulnerability and resilience.

### 6.1. Implications for Intervention

The emotion–feeling framework underscores that interventions must target the relational processes that bridge embodied reactivity and conscious experience. Parenting programs such as PCIT and Circle of Security enhance attunement and reflective functioning, thereby stabilizing synchrony at the emotion level while strengthening symbolic scaffolds for feeling [115,116,117,118]. Parent–Child Interaction Therapy (PCIT) offers a strong evidence base for improving emotional reciprocity and dyadic regulation in parent–child relationships. Originally developed for externalizing disorders, PCIT has since been adapted for children with autism spectrum disorder (ASD) and other developmental disabilities, demonstrating improvements in both behavioral and relational domains. Recent evidence highlights that PCIT not only reduces disruptive behaviors but also strengthens attachment security, caregiver sensitivity, and synchrony during interactional exchanges [119]. Such adaptations emphasize developmental attunement—slowing the pace of interaction, scaffolding communication, and enhancing emotional mirroring—allowing parents to respond more effectively to subtle cues of affect and engagement. These mechanisms make PCIT particularly valuable for neurodevelopmental populations where emotional reciprocity may be limited, underscoring synchrony as a critical pathway through which parent-mediated interventions exert their regulatory effects. Moreover, neuroscience-informed approaches—including HRV biofeedback, mindfulness-based parenting, and gaze-contingency feedback—directly modulate synchrony systems, demonstrating that interventions can be designed to align physiology and behavior in ways that facilitate the transition from arousal into reflective awareness [120,121,122]. These strategies are particularly critical for highly sensitive children, who stand to benefit disproportionately from supportive scaffolding (vantage sensitivity), and for those with CU traits or neurodevelopmental conditions, where compensatory interventions can restore synchrony and enable feeling construction. At the broader ecological level, policies that reduce parental stress, improve social support, and provide accessible early mental health care indirectly enhance synchrony by increasing caregivers’ emotional availability [123]. Developmental timing is also crucial: interventions in infancy emphasize physiological and attentional synchrony, while those in adolescence may focus on scaffolding autonomy, reflective dialogue, and intersubjective negotiation rather than pure physiological alignment [124].

### 6.2. Limitations

Several limitations of the current literature on parent–child synchrony and affective development should be acknowledged. One concern is publication bias: studies reporting significant synchrony effects (e.g., robust correlations between parent–child physiology or neural activity) are more likely to appear in the published record than those with null or inconsistent findings. This may create an overly optimistic picture of the strength and consistency of synchrony effects. Another important limitation is the over-reliance on WEIRD (Western, Educated, Industrialized, Rich, Democratic) populations. Most studies have been conducted in Western or high-income contexts, raising questions about generalizability. As noted in the cultural variability section, patterns of parent–child interaction and emotion socialization differ across cultural settings, and a “lack” of synchrony in one cultural context may reflect alternative caregiving norms rather than developmental deficits. Finally, many investigations are based on small sample sizes and cross-sectional designs, which limit inferences about developmental trajectories. Future research would benefit from larger, longitudinal, and more culturally diverse samples to evaluate how synchrony processes unfold across development. Highlighting these issues underscores that claims about parent–child emotion–feeling dynamics must be continually tested and refined to ensure they are both robust and inclusive of diverse human experiences.

### 6.3. Future Research Directions

Looking ahead, several future research directions emerge from this review that can further elucidate the development of emotion and feeling in parent–child dyads. First, there is a clear need for more longitudinal and cross-lagged studies tracking children over time. Most available evidence is cross-sectional, providing snapshots of different ages or dyads. Longitudinal designs—following the same parent–child pairs from infancy through later childhood—would allow researchers to directly observe how early emotional synchrony (or its absence) cascades into later outcomes. Such studies could answer, for instance, when and how initial emotion–feeling integrations (e.g., a toddler learning to label their fear with a parent’s help) forecast the child’s emotional competence or mental health years down the line. Cross-cultural research is another priority. As noted, feelings are culturally shaped and parenting practices around emotion socialization vary widely. Comparative studies across different cultural or socio-economic contexts will help determine which aspects of the emotion-to-feeling developmental pathway are universal (rooted in biology) and which are culture-specific (shaped by norms and belief systems) [124,125,126].

In addition, future research should strive to integrate multiple levels of analysis to capture the full complexity of parent–child emotional dynamics. Recent advances (e.g., dual-brain hyperscanning with fNIRS or EEG) make it possible to simultaneously record neural activity in parent and child during real interactions. Applying these tools more widely could illuminate real-time bi-directional processes—for example, how a mother’s brain and her toddler’s brain become synchronized when sharing an emotional moment, and how such neural coupling might predict the child’s emerging capacity for empathy or self-regulation. Likewise, incorporating physiological measures (beyond cortisol and heart rate, to include indices like oxytocin, skin conductance, or respiration) could deepen our understanding of emotion transmission. A multi-method approach—combining behavioral observations, self-reports (when children are old enough), neural data, and physiological data—will provide a more holistic picture of how emotion and feeling co-develop within relationships [127,128,129,130].

Another important direction concerns resilience and individual differences. Our review highlighted that some children (for instance, those high in sensory processing sensitivity) are disproportionately influenced by the quality of emotional input—flourishing under supportive conditions and struggling under adversity. Future work should explore the protective factors that enable resilient trajectories even among high-risk groups. For example, are there cases where children with a difficult temperament still develop strong emotion–feeling integration due to exceptionally supportive parenting or other environmental buffers? Unpacking these cases could inform tailored interventions that bolster protective processes. Similarly, investigating gender differences (if any) in emotion–feeling development, or how siblings might differentially negotiate these processes, could add nuance to our understanding of individual variability in normative pathways [130].

Finally, this field is poised to inform and benefit from applied intervention research. The distinction between emotion and feeling underscores that simply managing a child’s behavior (emotion expression) is not enough—caregivers and clinicians should also aim to foster the child’s understanding and integration of those emotions into feelings. Parent-focused interventions that encourage sensitive attunement, emotion labeling, and contingent responsiveness are likely to help children build robust emotion–feeling bridges. Indeed, preliminary studies with at-risk children show promise: for instance, interventions training parents in warm, explicit emotion communication have partially normalized attention to emotional cues and improved emotional understanding in children with callous–unemotional traits [130]. Future trials could extend such approaches to other groups (e.g., children with autism spectrum disorder who might benefit from augmented multimodal emotional cues) and examine long-term outcomes. In parallel, preventive programs in early childhood—such as attachment-based parenting programs or emotion-coaching curricula in preschools—should be tested for their effectiveness in enhancing emotional synchrony and the early emergence of feelings. Ultimately, an integrative developmental framework can guide interventions to target the relational mechanisms that transform emotion into feeling. By bolstering synchrony in attention, neural responses, and physiology, and by empowering parents to scaffold children’s emotional experiences with language and empathy, we can promote healthier emotional development. In summary, future research and practice should jointly focus on filling key knowledge gaps (through longitudinal, cross-cultural, and multi-level studies) and translating insights into strategies that support children’s journey from raw emotion to reflective feeling—laying a foundation for emotional well-being and resilience across the lifespan [131].

## 7. Discussion

The synthesis presented in this review has important clinical and social implications. By distinguishing emotion as an embodied system of action readiness and interpersonal signaling, and feeling as its subjective and symbolic correlate, developmental science can move beyond longstanding conceptual ambiguity to inform applied practice. This distinction matters clinically, as many interventions currently target “emotions” and “feelings” interchangeably, potentially diluting their effectiveness. Recognizing that dyadic synchrony provides the bridge between biological reactivity and subjective experience highlights the parent–child relationship as a central therapeutic leverage point. From a clinical standpoint, this framework underscores the need to design interventions that directly enhance attentional, neural, and physiological synchrony. Eye-tracking-guided parent–child tasks, biofeedback protocols, and parent-focused stress regulation training may amplify attunement and buffer against dysregulation in children at risk for emotional and behavioral difficulties. For populations with neurodevelopmental disorders or callous–unemotional traits, such targeted approaches may prove especially important in fostering adaptive emotional development.

The environmental sensitivity framework further refines these implications. Children differ in how strongly they respond to synchrony processes, with those high in sensory processing sensitivity or related susceptibility traits showing disproportionate outcomes depending on caregiving quality. In supportive environments, their heightened reactivity facilitates richer co-regulation and more differentiated feelings; in adverse environments, the same sensitivity accelerates dysregulation and fragmentation of emotional experience. Clinically, this implies that tailoring interventions to children’s sensitivity profiles—whether through vantage sensitivity or differential susceptibility models—can optimize effectiveness by matching strategies to developmental context. Socially, these insights emphasize the broader relevance of relational contexts in supporting resilience. Policies that strengthen early caregiving, reduce parental stress, and promote inclusive technologies can help create environments where children’s biological sensitivities are scaffolded into opportunities for growth rather than vulnerabilities for psychopathology. The recognition that environmental sensitivity moderates developmental trajectories also underscores the urgency of addressing inequities in caregiving resources, as these disparities disproportionately affect the most susceptible children.

Overall, the clinical and social significance of the current framework lies in its ability to link basic neurocognitive and psychophysiological research with practical pathways to intervention, prevention, and policy. By treating the dyad as the crucible of affective development, and by recognizing how environmental sensitivity modulates the movement from emotion to feeling, clinicians, educators, and policymakers can adopt a relational and context-sensitive approach to fostering well-being across diverse developmental and cultural settings.

## Figures and Tables

**Table 1:** Definitional distinctions between emotion and feeling based on classic and recent theories.

Feature	Emotion	Feeling
Core definition	A rapid, coordinated psychophysiological response to a salient stimulus, encompassing autonomic, hormonal, and behavioral reactions [3,12].	The conscious, subjective experience of an emotional state—the introspective awareness of bodily and neural changes [6,7,8].
Neural basis	Largely subcortical circuits, including amygdala, hypothalamus, and brainstem, supporting fast survival-oriented responses [9].	Cortical integration of interoceptive and bodily signals in the insula, anterior cingulate, and prefrontal cortices, enabling conscious awareness [8,10].
Consciousness	Can occur outside awareness; emotions involve automatic and unconscious bodily reactions [3,4].	By definition conscious; feelings are the subjective experience of emotion [6,9].
Temporal dynamics	Rapid-onset, short-lived responses aligned with external stimuli, often emerging within milliseconds [3].	Slower to arise, requiring cortical processing, and can persist over time through conscious awareness and reflection [8,13].
Measurement	Inferred from observable indices such as facial/vocal expressions, psychophysiological responses (heart rate, cortisol, fMRI, EEG), and behavior [12].	Primarily assessed via self-report, interviews, or rating scales reflecting subjective experience [4,13].
Ontogeny/Evolution	Emerges early in development and is evolutionarily ancient; even infants and animals display basic emotional responses [3,12].	Develops later with higher cognition, language, and self-awareness; refinement of reported feelings increases across childhood and adolescence [6,14].

## Data Availability

Not applicable.

## Abbreviations

The following abbreviations are used in this manuscript:

SPS	Sensory Processing Sensitivity
CU traits	Callous-unemotional traits
ADHD	Attention Deficit Hyperactivity Disorder
